# Data-driven mechanistic analysis of digital therapeutic–assisted training and evaluation of personalized protocol effects in children with attention deficit/hyperactivity disorder

**DOI:** 10.3389/fpsyt.2026.1794425

**Published:** 2026-06-19

**Authors:** Seon-Chil Kim, Na-Yeong Kong, Sun-Young Lee, Sang-Woo Lee

**Affiliations:** 1 Department of Biomedical Engineering, School of Medicine, Keimyung University, Daegu, Republic of Korea; 2 Department of Psychiatry, School of Medicine, Keimyung University, Daegu, Republic of Korea; 3 Senior Researcher, Biomedical Engineering Laboratory, Department of Biomedical Engineering, School of Medicine, Keimyung University, Daegu, Republic of Korea; 4 InTheTech Inc., Daegu, Republic of Korea

**Keywords:** ADHD, CAT, DTx, K-ARS, speed-accuracy regulation

## Abstract

**Introduction:**

Digital therapeutics (DTx) for children with ADHD have gained attention as adjunctive interventions to pharmacotherapy; however, evidence remains limited regarding the mechanisms underlying training performance decline and the clinical effectiveness of performance data–driven personalized protocols. This study aimed to identify the core mechanisms of performance decline using digital training performance data and to compare the effects of a mechanism-based personalized protocol with those of a standard protocol.

**Methods:**

A total of 40 children with ADHD (aged 5–12 years), diagnosed according to DSM-5 criteria, were randomly assigned to an experimental group (personalized protocol, n = 20) or a control group (standard protocol, n = 20). During week 1, all participants completed the same 10 training contents, from which accuracy, reaction time, and error types (commission and omission errors) were extracted to construct individualized performance profiles. During weeks 2–4, the experimental group received a personalized protocol in which content composition, difficulty, and feedback were adapted based on baseline performance profiles, whereas the control group followed a standard protocol. Total training volume was controlled to be identical between groups. Pre–post assessments were conducted using the Comprehensive Attention Test (CAT) sensitivity indices and the Korean ADHD Rating Scale (K-ARS). Baseline-adjusted ANCOVA and effect sizes were reported.

**Results:**

Performance data analyses indicated that performance decline was more closely associated with commission errors (impulsivity) than with omission errors, and a speed–accuracy trade-off was observed, whereby faster responses were associated with increased error rates.

**Discussion:**

These findings suggest that performance decline in digital training among children with ADHD may be related to impulsivity and speed–accuracy control characteristics. Furthermore, performance data–driven personalized DTx demonstrated greater improvements in objective attentional control and clinical symptoms than a standard protocol with an equivalent training volume.

**Clinical Trial Registration:**

https://cris.nih.go.kr, identifier KCT0010850.

## 1 Introduction

The diagnostic rate of pediatric attention deficit/hyperactivity disorder (ADHD) has been continually increasing in recent years due to improved access to child and adolescent psychiatric services and the development of standardized assessment tools (1). Pediatric ADHD is characterized by inattention, hyperactivity, and impulsivity; however, these core symptoms are frequently accompanied by various comorbid conditions, including anxiety and mood disorders as well as learning disabilities. Consequently, diagnoses based solely on a single-symptom scale are limited (2). Clinical diagnosis is typically based on behavioral observations and a multidimensional assessment approach that includes interviews and questionnaires administered to parents or caregivers (3).

ADHD is one of the most diagnosed neurodevelopmental disorders in childhood and is primarily attributed to genetic factors and imbalances in neurotransmitter systems, with symptom onset typically occurring before the age of 12 (4). Difficulties related to inattention and impulsivity during childhood may lead to poor academic achievement and reduced learning motivation, which often persist into adulthood, negatively affecting self-esteem, social relationships, and occupational functioning (5). Therefore, early ADHD diagnosis and treatment strategies aimed at long-term functional improvement are critical.

Currently, the primary treatment approach for ADHD is pharmacotherapy, which mainly involves administering central nervous system stimulants such as methylphenidate and non-stimulant medications such as atomoxetine (6). However, such pharmacological treatment is associated with potential adverse effects, including appetite suppression and sleep disturbances. Moreover, long-term use of these treatments may exert associated psychological burdens on both children and their caregivers (7). Behavioral therapy and cognitive training are often employed as adjunctive treatments. However, these approaches require sustained caregiver involvement and exhibit considerably varying treatment outcomes depending on individual differences (8). Against this backdrop, digital therapeutics (DTx) have recently gained attention as complementary ADHD treatment approaches (9, 10). DTx enable continuous intervention without temporal or spatial constraints and can be utilized as adjunctive treatment options without the risk of medication-related side effects (11). In particular, game-based or interactive content-driven DTx have been proposed as novel intervention options for pediatric ADHD since they can enhance engagement and adherence among children.

However, the clinical effectiveness of DTx is determined not only by whether the intervention is used but also by the content design strategies and cognitive and behavioral responses elicited during task performance (12). Conventional digital training content has primarily been structured either in question–answer (quiz-based) formats centered on correct response selection or in game-based formats emphasizing situation-specific problem solving (13). Although game-based content may be motivating and engaging, the complexity of visual elements and screen composition may paradoxically induce attentional distraction or impulsive responses in children (14). Contrastingly, quiz-based structures are relatively amenable to individualized configurations; however, their ability to adequately capture changes in behavioral regulation that emerge during actual task performance is reportedly limited (15). Most existing digital intervention content is delivered using uniform content structures and linear difficulty progression based on group averages, thereby limiting the ability to design interventions that account for individual differences in symptom profiles and performance patterns among children (16, 17).

These limitations may stem from an insufficient mechanistic understanding of core ADHD pathological characteristics and the lack of a systematic framework for translating such understanding into content design. Performance impairments in children with ADHD are influenced by both inattention and, substantially, impulsivity (18, 19). In digital environments, rapid decision-making and immediate responses are frequently required, and failures in regulating response speed can increase commission errors, thereby significantly contributing to reduced accuracy (20). Prior digital intervention studies and content designs have largely focused on enhancing attention, whereas approaches that quantitatively analyze impulsivity regulation mechanisms and incorporate them into performance-based intervention programs remain limited (21).

Furthermore, children with ADHD exhibit substantial inter-individual variability in performance patterns depending on age, cognitive level, and symptom presentation, indicating clear limitations in uniformly applying identical digital training protocols to all individuals (22). To leverage digital content aligned with therapeutic objectives effectively, it is necessary to analyze individual characteristics based on behavioral performance metrics measured during task execution—such as accuracy, response time, and error types—and design personalized training protocols accordingly. Particularly, distinguishing impulsivity and attentional control patterns through the relationship between response speed and error type may provide critical insights for enhancing digital intervention precision.

Accordingly, this study aimed to analyze the behavioral indicators (accuracy, response time, and error types) observed during DTx adjunct training in children with ADHD. Specifically, the objectives were as follows: First, we sought to empirically determine whether performance impairments are predominantly driven by inattention or impulsivity. Second, we aimed to analyze the speed–accuracy regulation mechanism that explains performance characteristics in children with ADHD. This was achieved by examining the relationship between response time and error types. Further, we sought to evaluate the impact of impulsivity on task outcomes in digital environments. Third, based on digital performance data, we aimed to classify performance patterns in children with ADHD and derive user-type–specific cognitive and behavioral characteristics, thereby constructing mechanism-based personalized DTx adjunct training protocols. Finally, by comparing an experimental group that received personalized protocols with a control group that received standard (non-personalized) protocols, we aimed to verify the clinical effectiveness of personalized digital interventions. Through this design, the present study aimed to interpret the operational mechanisms of ADHD digital interventions based on digital training log data and to propose content combinations and stepwise implementation strategies aligned with therapeutic objectives. Ultimately, this study sought to contribute to the clinical application of DTx and the establishment of evidence-based guidelines for personalized content design.

## 2 Methods

### 2.1 Study participants

The study participants were children aged 5 to under 12 years diagnosed with ADHD according to the diagnostic criteria of the Diagnostic and Statistical Manual of Mental Disorders, Fifth Edition (DSM-5) (23, 24). The inclusion criteria were as follows: provision of written informed consent by a legal guardian and assent from the child; ability to comply with the evaluation and intervention procedures as determined by the principal investigator; and normal intellectual functioning as assessed using the Korean version of the Wechsler Intelligence Scale for Children–Fifth Edition (K-WISC-V) (25). Exclusion criteria included the presence of medical, psychiatric, or neurological conditions other than ADHD; physical conditions that significantly limited the use of digital devices; marked motor impairments, including seizure disorders, color vision deficiency; enrollment or concurrent participation of a family member in the same study; and any other condition deemed inappropriate for study participation by the investigator.

In the study design stage, we determined a 40-participant target sample size based on prior literature and the practical feasibility of recruitment. Instead of a formal *a priori* sample size calculation, we performed an *a priori* sensitivity analysis using G*Power version 3.1.2 to assess the adequacy of the target sample size (26). Accordingly, under a two-tailed significance level of α = 0.05 and statistical power of 1–β = 0.80, we estimated the minimum detectable effect size (MDE) for an independent two-group comparison at Cohen’s d ≈ 0.96. For an analysis of covariance (ANCOVA), assuming a baseline–posttest correlation of r ≈ 0.60, the minimum detectable effect size was estimated as d ≈ 0.77 (corresponding to f ≈ 0.39).

Forty-two children were screened for eligibility, of whom two were excluded because they did not meet the inclusion criteria. The remaining 40 participants were enrolled and randomly assigned to one of two groups: an experimental group receiving a personalized DTx protocol combined with ongoing pharmacotherapy (n = 20) and a control group receiving a standard DTx protocol with linear difficulty progression combined with pharmacotherapy (n = 20). None of the participants dropped out during the follow-up period and all 40 enrolled participants (20 per group) were included in the final analysis. While the participants were allowed to continue taking their pre-existing ADHD medications throughout the study period, they could not begin new medications during this period. Permitted concomitant medications included methylphenidate- and atomoxetine-based agents, which were maintained within the prescribed regimens established before study enrollment.

**Figure 1:**
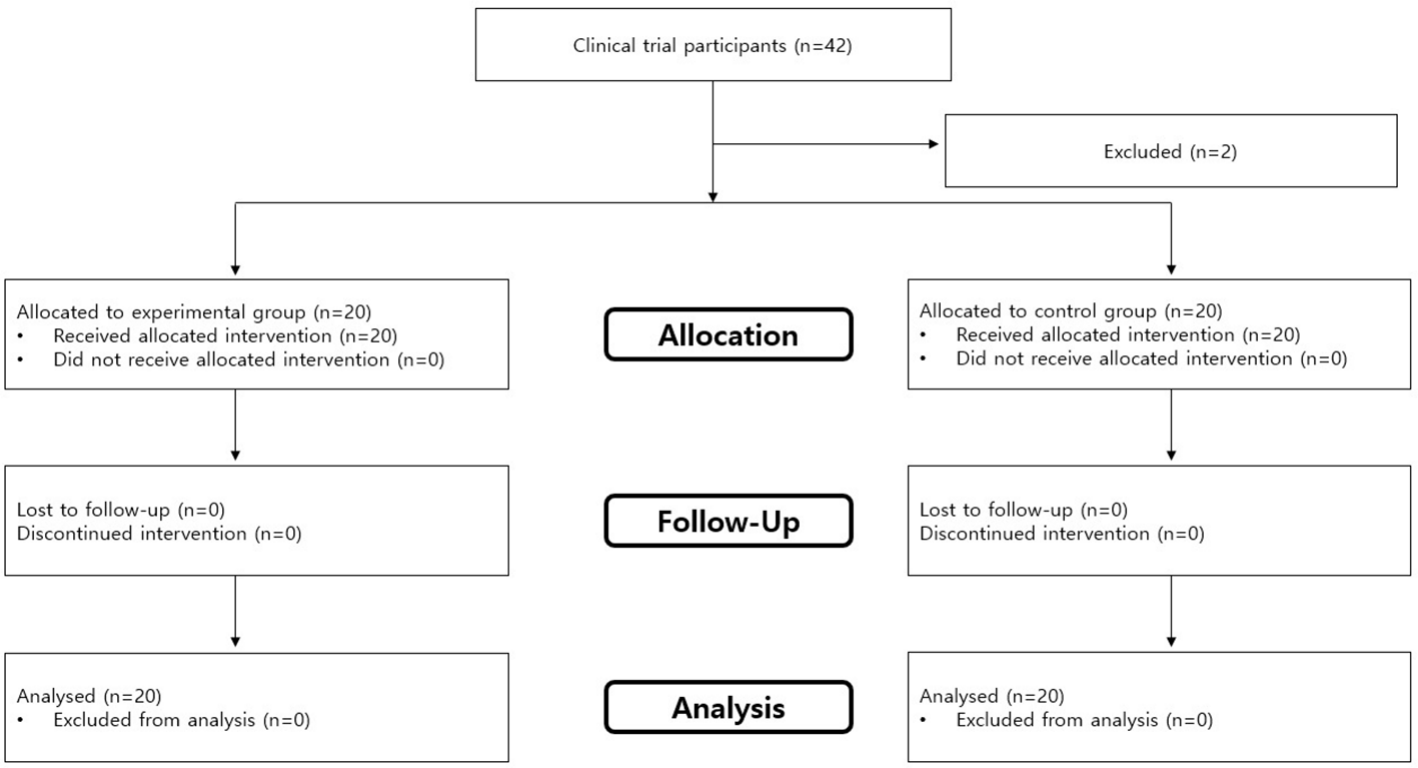
Flow diagram of study participants.

Baseline ADHD symptom severity was comparable between the two groups. Prior to the intervention, symptom severity was assessed using the Clinical Global Impression–Severity (CGI-S) scale, an observer-rated measure widely used to evaluate illness severity and clinical course. Beside age and sex distributions, the two groups were generally similar in major demographic and baseline clinical characteristics, including baseline symptom severity. **Figure 1** depicts the study design.

### 2.2 Study ethics

We conducted this study prospectively at the Department of Psychiatry of Keimyung University Dongsan Hospital from May 26, 2025, to September 30, 2025 (four months). Prior to data collection and assessment, we obtained ethical approval from the Institutional Review Board (IRB No. 2025-03-002-003) and registered the study with the Clinical Research Information Service (CRIS No. KCT0010850).

To facilitate their informed consent, we provided the participants and their legal guardians information detailing the study objectives, participation procedures, potential risks and benefits, confidentiality policy, and assurance that the collected data would be used solely for research purposes. Baseline data collection and experimental procedures commenced only after receiving written informed consent.

We selected participants from among applicants based on a comprehensive evaluation that included clinical interviews conducted by board-certified psychiatrists as well as psychological assessments, such as attention and intelligence tests. Although we did not anticipate that the participating children would experience direct adverse effects, we acknowledged the possibility of discomforts associated with digital device use, such as frustration, headache, and dizziness. To minimize such risks, device usage time was monitored and restricted to less than 30 minutes per session through a built-in monitoring program.

### 2.3 Assessment tools and procedures

The digital intervention program provided to the participants consisted of two protocols: a personalized protocol for the experimental group and a standard protocol for the control group. Specifically, the experimental group received a protocol in which the content composition, difficulty level, feedback, and training schedule were reconfigured according to participant-specific profiles derived from individual performance patterns, including accuracy, response time, and error types. The control group was provided with a standard protocol using the same content library, delivered according to a fixed sequence and predefined rules of linear difficulty progression, without differentiation by user type.

During the first week, both groups completed the same set of ten content modules to assess baseline performance characteristics. Subsequently, we conducted training according to group-specific protocols over Weeks 2–4 (three weeks). For the experimental group, we constructed and applied individualized training programs based on analyses of baseline performance data obtained during Week 1, including individual accuracy profiles, response time characteristics, and error patterns (e.g., omission and commission errors). Based on these analyses, content composition, difficulty levels, and feedback were tailored to each child’s performance type. In contrast, the control group used the same content library, but completed a standard program delivered in a fixed sequence with predefined rules for linear difficulty progression, without individual performance-based stratification, over the same three-week period. Total training exposure, including cumulative training time and exposure metrics (number of sessions and task screens), was matched between the two groups to allow for an independent comparison of personalization effects.

To evaluate the effectiveness of personalized digital content in DTx interventions, both groups underwent pre- and post-intervention assessments. The primary outcome variables of this study were changes in objective attention-related measures and changes in clinical symptom severity. Specifically, to assess changes in attention in children with ADHD objectively, the Comprehensive Attention Test (CAT) was administered at baseline and post-intervention. The CAT is a computerized performance-based assessment in which age-appropriate subtests are selectively applied. We used four subtests to evaluate selective attention and inhibitory control: Visual Selective Attention Task, Auditory Selective Attention Task, Flanker Task, and Sustained Attention to Response Task (27). Since this study aimed to interpret performance impairments in children with ADHD from the perspectives of impulsivity and selective attention regulation and to use these interpretations as the basis for personalized protocol design, we selected the sensitivity index—reflecting the ability to discriminate target from non-target stimuli—as the primary performance metric (28). The sensitivity index enables the quantitative assessment of stimulus discrimination and inhibitory control while minimizing the influence of response bias, making it suitable for analyses that differentiate content composition, difficulty, and feedback according to individual performance characteristics.

Clinical symptom severity was assessed using the Korean ADHD Rating Scale (K-ARS), which was completed by caregivers (parents). The K-ARS is an 18-item scale comprising two subdomains: inattention and hyperactivity–impulsivity. Each item is rated on a 4-point Likert scale ranging from 0 (not at all) to 3 (very often), yielding a total score range of 0–54 (29). In this study, both the total K-ARS score and the percentage change in the score were used as primary indicators of clinical symptom changes. The percentage change was calculated as {(Baseline K-ARS − 4-week follow-up K-ARS)/Baseline K-ARS} × 100. The reliability and validity of the K-ARS have been established in previous studies (30). While the CAT captures objective, performance-based changes in attention and inhibitory function, the K-ARS reflects changes in symptom severity observed in daily life; therefore, both instruments were used in combination to comprehensively evaluate the effects of the digital intervention.

Randomization was performed by an independent external contract research organization (CRO), and allocation concealment was maintained such that the investigators were unaware of group assignments at the time of participant enrollment. However, due to the nature of the digital therapeutic intervention, blinding of participants and their caregivers was not feasible, as they could recognize the assigned intervention condition after allocation. In addition, investigators became aware of group assignments following randomization in order to administer the intervention, and therefore full blinding of the investigators and analysts was not possible. Accordingly, while allocation concealment was ensured, blinding was only partially implemented, and the potential for bias arising from this limitation should be considered when interpreting the results.

### 2.4 Digital content types

**Figure 2:**
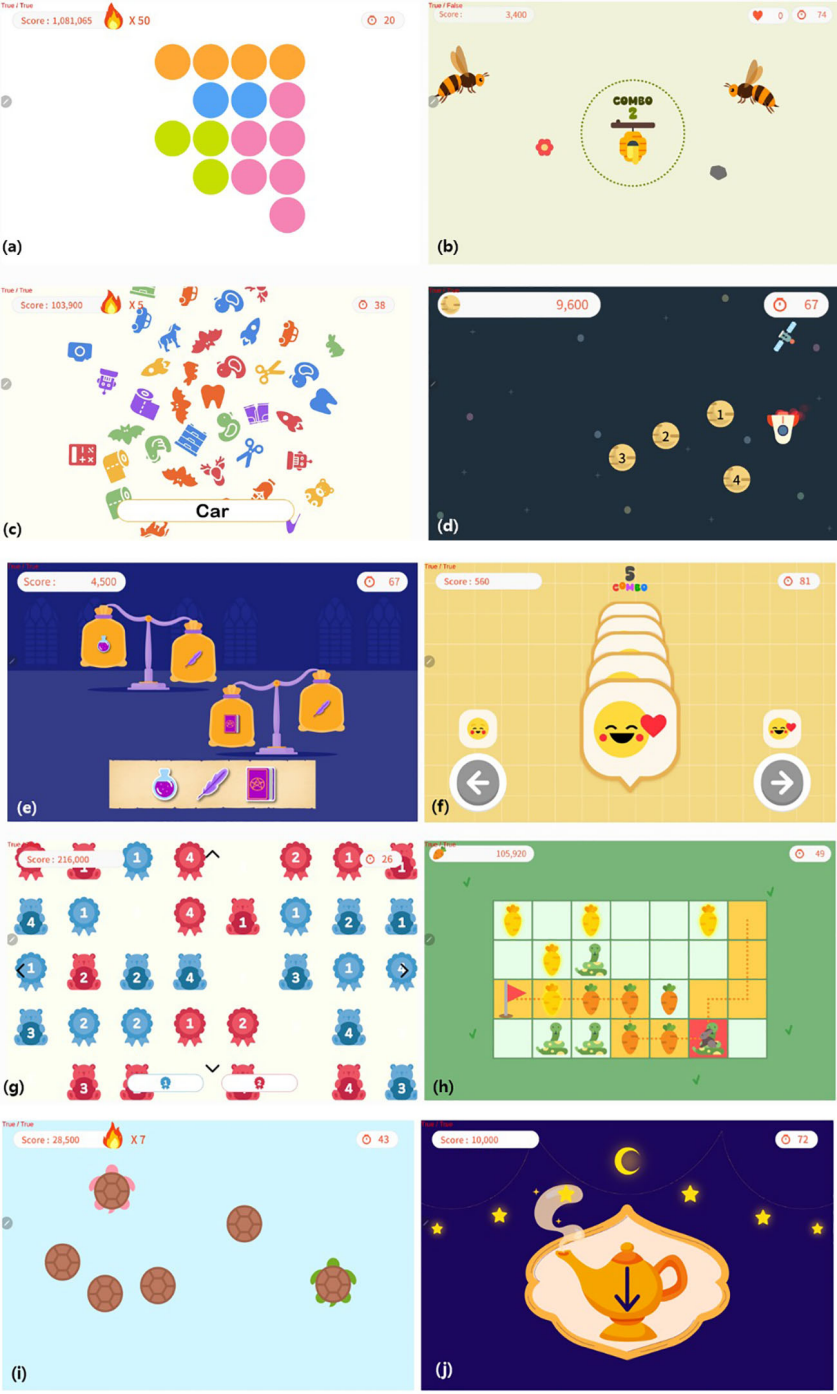
Major content screens: **(a)** Big: A task requiring participants to compare circles by color and sequentially select the colors starting from the least frequent. **(b)** Bee Protector: A selective response task in which participants are required to select flower stimuli, eliminate bees by touching them, and avoid distracting stimuli such as rocks. **(c)** Find Object: A task involving selecting all object stimuli of various colors that correspond to a keyword presented at the bottom of the screen. **(d)** Space Travel: A task requiring participants to connect paths while avoiding interfering spacecraft. **(e)** Scale Play: A weight-comparison task in which participants compare objects of different shapes presented on one or two balance scales and determine which object is heavier. **(f)** Choice Card: A classification task in which participants categorize facial expressions as positive or negative within a limited time. **(g)** Bear: A rule-based selection task in which participants choose an item matching the target color and shape displayed at the bottom of the screen, such that the selected number and the presented number sum to five. **(h)** Rabbit: A path-planning task in which participants create and maintain a route to allow a rabbit to reach and eat carrots. **(i)** Real Turtle: A visual tracking and working memory task in which two target turtles are initially presented with visible limbs. After the limbs disappear and multiple visually identical turtle shells begin to move, participants are required to identify and select the shells corresponding to the initially presented turtles. **(j)** Magic Lamp: A rhythm-based selective response task in which stars illuminate sequentially with auditory cues at varying tempos, while a directional arrow appears on the lamp. Participants are required to swipe the lamp in the indicated direction specifically when the highest central moon lights up.

The digital therapeutic intervention used in this clinical trial consisted of quiz-based game content implemented on the EYAS-Focus digital therapeutic platform (2024; Indertec Co., Ltd., Daegu, Republic of Korea). The digital content we used was developed for use on mobile devices and implemented in a user-friendly program environment to ensure ease of use during task execution. For both the experimental and control groups, the content used during the one-week baseline assessment phase to evaluate participant characteristics comprised ten distinct modules, as illustrated in **Figure 2**. The developed content was designed to capture performance characteristics related to accuracy and response time while also enabling the analysis of major error types.

The intervention content we used covered four categories: foundational content designed for initial adaptation and confidence building (two modules), focused training for impulsivity control (three modules), speed–accuracy trade-off training (three modules), and sustained search and perseverance training (two modules). Depending on the content type, each module was structured across ten difficulty levels, with the initial levels consisting of programs that required responses to approximately 30 stimuli. Through analysis of the final learning outcomes, changes in performance achievement (accuracy), response time, and error patterns were assessed. Accordingly, to construct personalized training content tailored to individual performance levels, we defined performance evaluation indices for each content category. The selected indices included the accuracy, response time, and error types obtained during the execution of multiple programs in the initial stages, which were used to characterize the individual performance profiles.

The first performance index, accuracy, was analyzed in relation to content difficulty settings and was used to identify relative performance characteristics across content types based on the observed correct response rates. Through this process, content associated with relatively low accuracy and that associated with high accuracy were distinguished, and the combination of difficulty level and performance pattern was used as a criterion for configuring individualized training content. The second index, response time, was analyzed by considering inter-individual variability across content types and examining its relationship with accuracy and error patterns. Certain content types exhibited performance patterns characterized by both rapid response times and low accuracy, reflecting prominent speed–accuracy trade-off effects (31). Based on these characteristics, such content was classified as requiring precise response regulation under rapid-response conditions.

Finally, we analyzed error types by examining the persistence and repetition of commission and omission errors to determine the presence of impulsivity-dominant or attention-deficit-dominant patterns. Based on this analysis, the proportion of training content targeting impulsivity inhibition and content designed to enhance sustained attention was adjusted and reconfigured accordingly.

### 2.5 Data analysis

Statistical analyses were performed using SPSS software (version 23.0; SPSS Inc., Chicago, IL, USA). All statistical tests were two-tailed and set at a 0.05 significance level (32). To evaluate clinical effectiveness, the primary analysis population was defined according to the modified intention-to-treat (ITT) principle, while a per-protocol analysis was conducted as a secondary analysis. The final interpretation of clinical outcomes was primarily based on the modified ITT analysis results (33). The modified ITT population included participants who were randomized, received the device equipped with the ADHD digital training program, and had data available for at least one primary efficacy outcome after baseline assessment. For the efficacy variables, no missing data occurred as all 40 enrolled participants completed the full study period with zero dropouts. Therefore, no data imputation was required. The per-protocol population consisted of participants within the modified ITT set who had no major protocol violations (e.g., premature withdrawal, violations of inclusion/exclusion criteria, or randomization violations) and who demonstrated treatment adherence of at least 80%.

We summarized the participants’ general characteristics using frequencies and percentages for categorical variables and means with standard deviations for continuous variables. The chi-square test was employed to assess the homogeneity of baseline demographic characteristics between the experimental and control groups. For baseline comparisons of dependent variables, we employed the Student’s t-test when normality assumptions were satisfied and the Mann–Whitney U test when normality was not met (34). Within-group pre–post changes in digital content performance were analyzed using paired t-tests and between-group differences in change scores were analyzed using independent t-tests. When normality assumptions were violated, the Wilcoxon signed-rank test and Mann–Whitney U test were used for within-group and between-group comparisons, respectively. Baseline scores were included as covariates to evaluate between-group differences in pre–post changes while adjusting for baseline values. Additionally, we conducted ANCOVA adjusted for baseline values to compare changes in the outcome measures after the four-week intervention period. This approach was used to control for potential baseline differences between groups and to estimate the effects attributable to digital content training more accurately. Furthermore, correlation analyses were conducted to explore the trends in performance indicators over the training period. In the experimental group, relationships between the number of days of digital content use and performance metrics—including score, accuracy, response time, commission errors (impulsivity-related errors), and omission errors (inattention-related errors)—were examined using Pearson’s correlation coefficients (35). These analyses assessed learning effects and temporal trends in performance patterns over the course of training.

Along with statistical significance, we considered effect sizes. For the paired-sample t-tests, we calculated Cohen’s d and found values of 0.2, 0.5, and 0.8, which were interpreted as small, medium, and large effects, respectively. For ANCOVA adjusted for baseline values, partial eta squared (η^2^) showed values of 0.01, 0.06, and 0.14, interpreted as small, medium, and large effects, respectively (36).

## 3 Results

### 3.1 Participant information

The demographic characteristics of the participants are presented in **Table 1**. Most of the participants in both groups were boys (16 [80%] for the experimental and 15 [75%] for the control). Further, the results showed no statistically significant between-group differences in sex distribution (χ^2^ = 0.55, *p* > 0.05) and age (t = 0.85, *p* > 0.05) between the two groups. No comorbidities were identified among the participants.

**Table 1:** Demographic information of test subjects.

Variable	Category	Experimental group (N = 20)		Control group (N = 20)		*P*-value	Accompanying symptoms
		Mean or N	SD or %	Mean or N	SD or %		
Gender	Boy	16	80	15	75	0.458*	None
	Girl	4	20	5	25		
Age		9.19	1.06	9.25	1.58	0.314**	None

* Chi-square test, ** Independent t-test.

### 3.2 Preliminary characteristic analysis for applying personalized content

#### 3.2.1 Evaluation results of basic content

**Figure 3:**
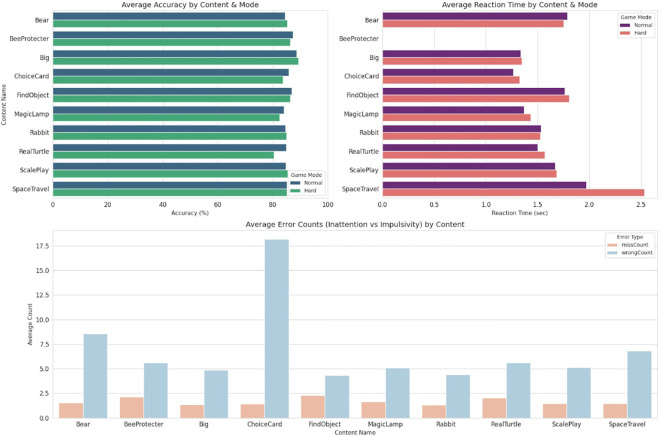
Performance evaluation results of the basic content.

Among the content provided to the 40 participating children, Big was identified as the easiest, showing the highest mean accuracy of 88.9%, whereas Magic Lamp exhibited the lowest mean accuracy of 83.5%, indicating a relatively higher level of difficulty. Regarding response time, Choice Card demonstrated the fastest mean response time (1.29 s); however, during actual implementation, its accuracy tended to decrease sharply owing to operational errors (wrong counts). This suggests that the task characteristics of this content require rapid decision-making, thereby inducing impulsive errors. Conversely, Space Travel showed the slowest mean response time (2.15 s), presumably reflecting the greater cognitive demand associated with complex visual search and decision-making processes. Error-type analysis categorized error types into impulsivity- and inattention-related errors. Choice Card showed a markedly higher proportion of impulsivity-related errors, indicating its potential suitability as impulsivity control training content. Contrastingly, Find Object exhibited a higher rate of inattention-related errors, suggesting that it requires sustained attention. These results are illustrated in **Figure 3**.

To construct an individualized, personalized training program, the characteristics of the ten content modules were classified based on their performance outcomes. Content modules such as Big and Bee Protector, which showed high accuracy and low task accessibility, were incorporated to facilitate early adaptation and enhance confidence during the initial training phase. Choice Card was selected as the focus impulsivity control training tool because it elicited the highest frequency of impulsive errors, making it suitable for inhibition reinforcement training. Conversely, content modules such as Magic Lamp demonstrated relatively fast response times accompanied by lower accuracy, reflecting a pronounced speed–accuracy trade-off inherent to the task. Owing to these characteristics, this content was utilized in training contexts that require accurate response regulation under time pressure. Finally, game modules such as Space Travel and Find Object, which required longer response times, were employed as patience and sustained attention training tools.

#### 3.2.2 Performance outcomes of individually personalized content

**Figure 4:**
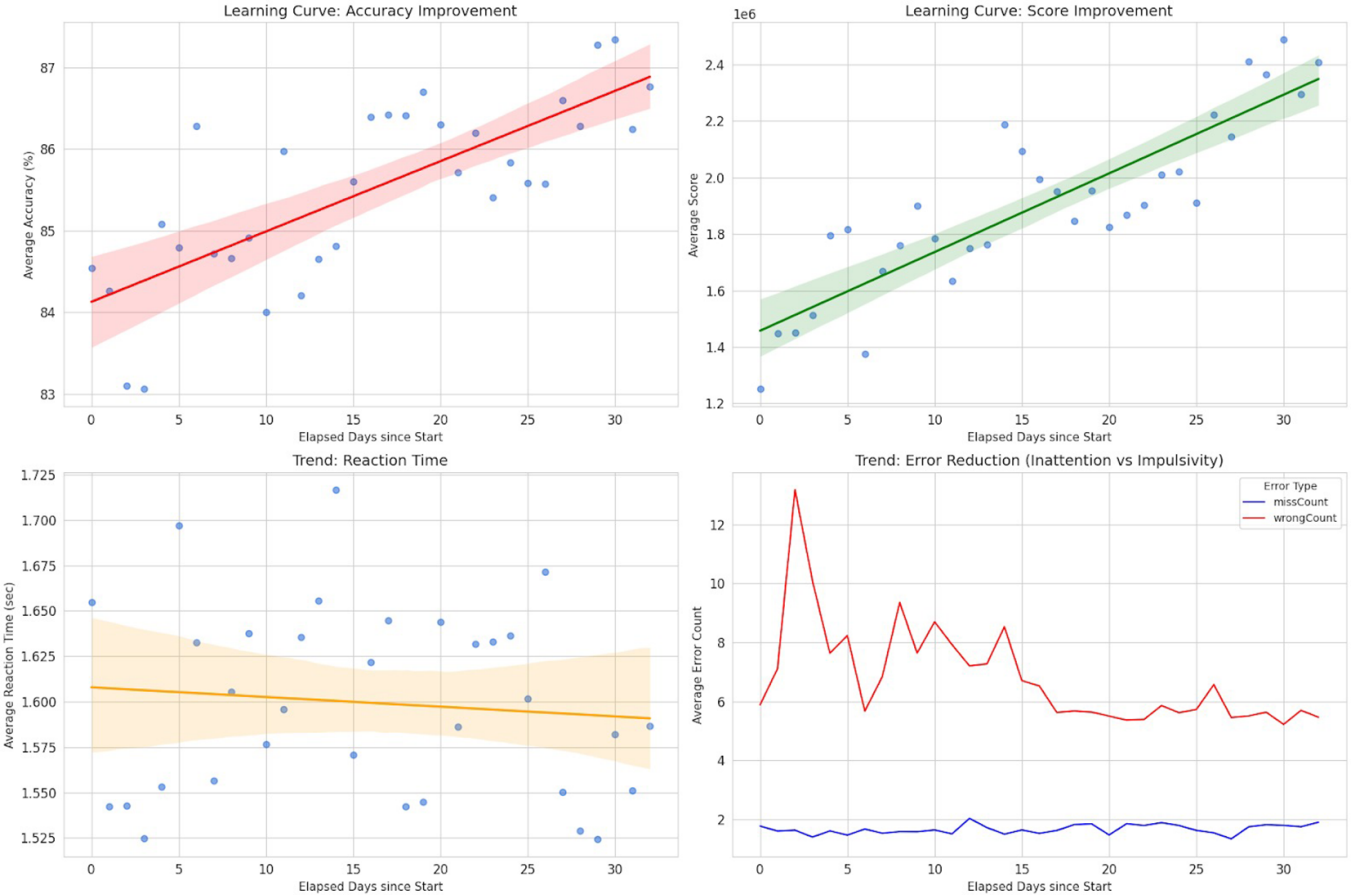
Changes in learning curves, response time, and error rates.

The 3-week performance outcomes of the personalized content provided to the experimental group were analyzed using learning-effect-based trend analysis. The pre-post changes in performance metrics of the experimental group are presented in **Table 2**. Changes in the children’s performance according to the training period (number of usage days), including achievement scores, response times, and error patterns are presented in **Figure 4**.

**Table 2:** Pre-post changes in performance metrics of the experimental group.

Metric	Pre mean	Post mean	Change	*P*-value	Result interpretation
Score	1,339,763	2,397,445	+1,057,682 (79%↑)	<0.001	Highly significant improvement
Accuracy (%)	84.47	86.78	+2.32%	0.004	Significant improvement
Impulsive Error (Wrong count)	7.01	5.46	−1.55 (22%↓)	0.140	Decreasing trend (not statistically significant)
Inattention Errors (Miss count)	1.68	1.82	+0.13	0.349	No significant difference
Response Time (s)	1.573	1.573	0.000	0.995	No change (stable performance)

The analysis of performance changes over the training period revealed a clear upward trend in achievement scores, correlating positively with training duration (r = 0.158). This indicates that as training progressed, the children became more familiar with the game rules and achieved higher performance scores. Accuracy exhibited a gradually increasing trend (r = 0.083), starting from an initial mean accuracy of approximately 84.5% and showing an overall upward tendency throughout the training period.

In contrast, response time remained relatively stable with minimal change (r = –0.006), suggesting that the training effect was characterized by maintaining a consistent and controlled response speed rather than promoting faster responses. Impulsivity-related errors showed a gradually decreasing trend (r = –0.062), with the reduction becoming more pronounced as the training duration increased.

Inattention-related errors remained low with minimal variation (r = 0.030) and demonstrated a slightly decreasing pattern over time. This suggests that after the initial adaptation phase, improvements in inhibitory control became more evident. Overall, as the training period increased, achievement scores improved while impulsive errors decreased, indicating a positive learning effect of the personalized training program.

In the personalized training group, pre–post significance testing of learning performance demonstrated a statistically significant improvement in accuracy (*p* = 0.004). This finding indicates that the observed performance gains were not limited to increased achievement scores, but were accompanied by improvements in the quality of task execution. In particular, we observed a positive trend in impulsivity-related indices. The mean number of impulsive errors (wrong counts) decreased from 7.01 to 5.46, representing an approximate 22% reduction; however, this change did not reach statistical significance (*p* = 0.140), which may be attributable to substantial inter-individual variability. We did not observe any statistically significant pre–post differences in response time or omission errors (miss counts). This suggests that the children’s accuracy improved without sacrificing response speed, which can be interpreted as a qualitative improvement in performance efficiency.

### 3.3 Clinical outcomes

#### 3.3.1 Comprehensive Attention Test (CAT)

**Figure 5:**
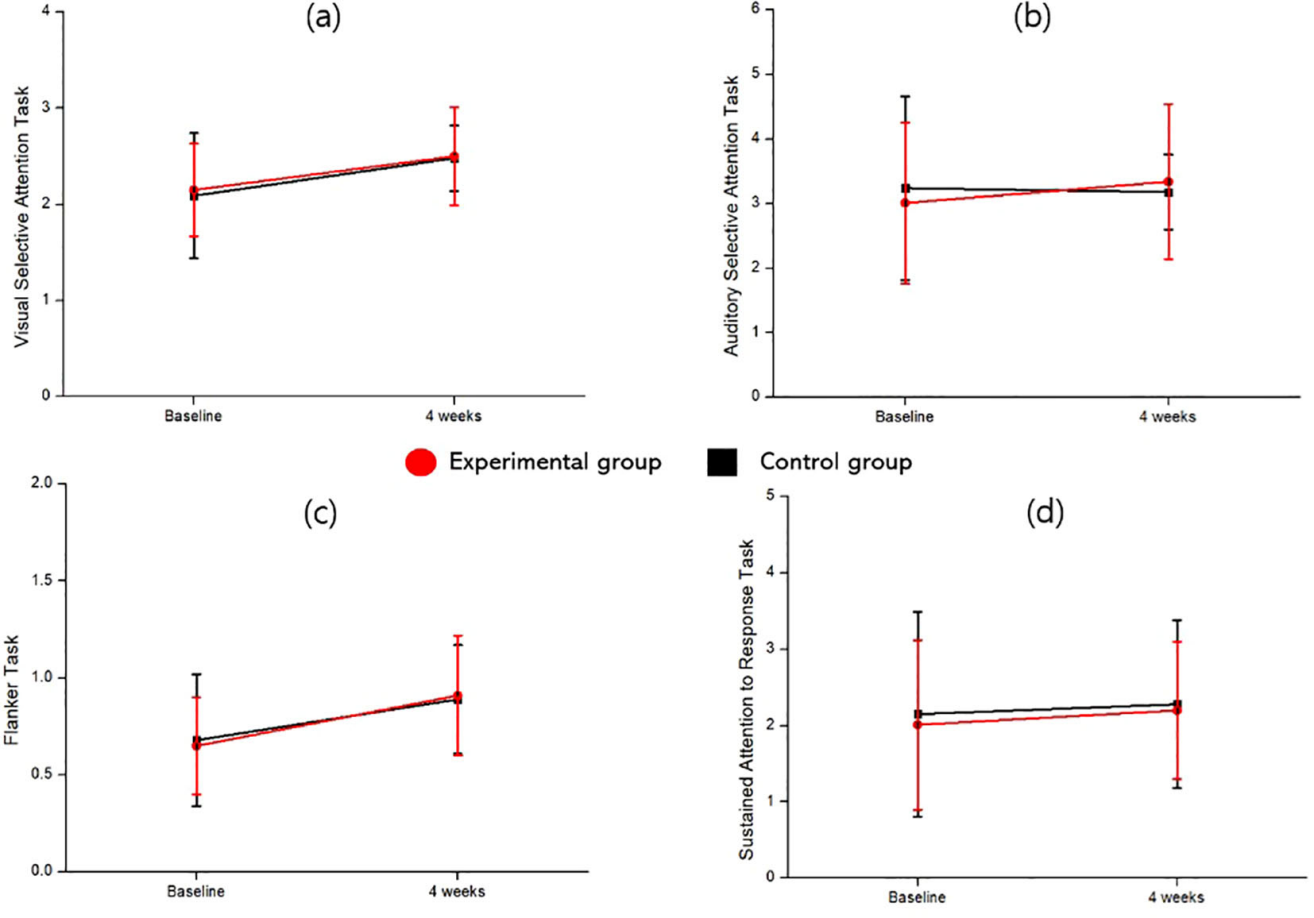
Attention task_sensitivity factor. **(a)** Visual selective attention task **(b)** Auditory selective attention task **(c)** Sustained attention to response task **(d)** Flanker task Error bars represent standard deviations (SD).

The results of the Attention Task Sensitivity Factor analysis derived from the CAT are presented in **Table 3** and **Figure 5**. The performance level scores of both the experimental and control groups exceeded 2 across all subtest domains, except the Flanker Task. In the Flanker Task domain, the experimental group showed a statistically significant increase in performance after four weeks compared with baseline (*p* = 0.038), accompanied by a large effect size (Cohen’s d = 0.93), indicating a clinically meaningful improvement. Furthermore, we observed a significant between-group difference in Flanker Task performance at the 4-week follow-up (*p* = 0.024), with the experimental group exhibiting higher performance levels than the control group.

**Table 3:** CAT; rate of change in attention sensitivity factor.

Instrument	Subscale/Task	Measure	Experimental group (N = 20)		Control group (N = 20)		*P*-value	Effect size
			Mean	SD	Mean	SD		
Attention Task_ Sensitivity Factor	Visual Selective Attention Task	Baseline	2.15	0.48	2.09	0.65	0.154*	
		4 weeks	2.50	0.51	2.48	0.34	0.285*	
		*p*-value	0.089**		0.548**		0.452***	0.015##
		Effect size	0.401#		0.137#			
		Rate of change (%)	16.3		18.7		0.254*	0.36#
	Auditory Selective Attention Task	Baseline	3.01	1.25	3.24	1.42	0.615*	
		4 weeks	3.34	1.20	3.18	0.58	0.088*	
		*p*-value	0.054**		0.245**		0.102***	0.071##
		Effect size	0.459#		0.268#			
		Rate of change (%)	11.0		1.9		0.085*	0.55#
	Flanker Task	Baseline	0.65	0.25	0.68	0.34	0.056*	
		4 weeks	0.91	0.31	0.89	0.28	0.024*	
		*p*-value	0.038**		0.240**		0.540***	0.010##
		Effect size	0.93#		0.68#			
		Rate of change (%)	40.0		30.9		0.040*	0.66#
	Sustained Attention to Response Task	Baseline	2.01	1.11	2.15	1.34	0.458*	
		4 weeks	2.20	0.90	2.28	1.10	0.650*	
		*p*-value	0.010**		0.043**		0.056*	0.090##
		Effect size	0.19#		0.11#			
		Rate of change (%)	9.45		6.05		0.044***	0.65#

*Independent t-test, **Baseline vs. 4wks paired t-test, ***Analysis of Covariance (ANCOVA) adjusted for baseline # Cohen’s d ## η^2^.

The rate-of-change analysis revealed a 40.0% improvement for the experimental group, which was significantly greater than the 30.9% improvement observed in the control group (*p* = 0.040), corresponding to a medium-to-large effect size (Cohen’s d = 0.66). In the Sustained Attention to Response Task domain, both groups demonstrated score improvements at four weeks compared with baseline; however, the experimental group’s rate of improvement was significantly higher (9.45%) than that of the control group (6.05%, *p* = 0.044). This difference also corresponds to a medium-to-large effect size (Cohen’s d = 0.65).

#### 3.3.2 Korean Version of the Attention-Deficit/Hyperactivity Disorder Rating Scale (K-ARS)

**Figure 6:**
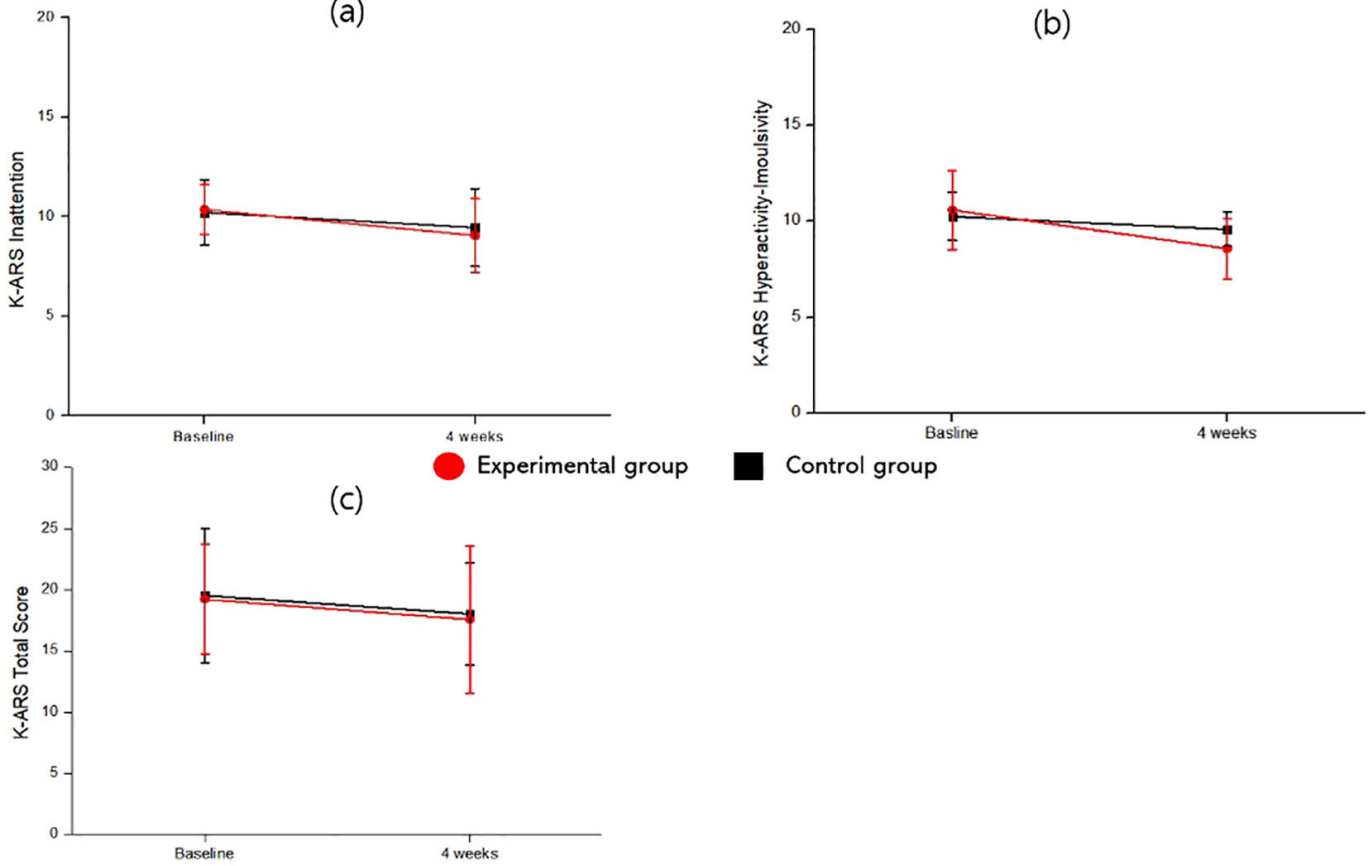
K-ARS score. **(a)** K-ARS Inattention **(b)** K-ARS hyperactivity–impulsivity **(c)** K-ARS Total Score Error bars represent standard deviations (SD).

The total, inattention, and hyperactivity–impulsivity subscale scores along with their rates of change on the K-ARS are presented in **Table 4** and **Figure 6**. The experimental group showed a statistically significant reduction in hyperactivity–impulsivity scores at four weeks compared with baseline (*p* = 0.046). After adjusting for baseline scores as a covariate, ANCOVA revealed that the experimental group exhibited a significantly greater reduction at four weeks than the control group (*p* = 0.025). This difference corresponded to a moderate-to-large effect size (η^2^ = 0.129), suggesting that the personalized DTx program had a meaningful effect on alleviating hyperactivity–impulsivity symptoms. Further, the K-ARS total score demonstrated a significantly greater reduction in the experimental group than in the control group four weeks after baseline adjustment (*p* = 0.040). This difference was associated with a moderate-to-large effect size (η^2^ = 0.109), indicating that the program also had a positive impact on overall ADHD symptom improvement.

**Table 4:** Rate of change in the K-ARS total score.

Instrument	Subscale/Task	Measure	Experimental group (N = 20)		Control group (N = 20)		*P*-value	Effect size
			Mean	SD	Mean	SD		
ADHD Rating	K-ARS Inattention	Baseline	10.35	1.25	10.20	1.65	0.205*	
		4 weeks	9.05	1.85	9.45	1.95	0.120*	
		*p*-value	0.064**		0.150**		0.518***	0.011##
		Effect size	0.44#		0.34#			
		Rate of change (%)	−12.6		−7.4		0.670*	
	K-ARS Hyperactivity-Impulsivity	Baseline	10.58	2.05	10.25	1.25	0.548*	
		4 weeks	8.58	1.58	9.58	0.89	0.340*	
		*p*-value	0.046**		0.251**		0.025***	0.129##
		Effect size	0.477#		0.265#			
		Rate of change (%)	−18.9		-6.5		0.102*	
	K-ARS Total Score	Baseline	19.24	4.48	19.54	5.48	0.108*	
		4 weeks	17.58	6.02	18.04	4.20	0.201*	
		*p*-value	0.205**		0.310**		0.040***	0.109##
		Effect size	0.293#		0.233#			
		Rate of change (%)	−8.6		−7.7		0.520*	

*Independent t-test, **Baseline vs. 4wks paired t-test, ***Analysis of Covariance (ANCOVA) adjusted for baseline # Cohen’s d ## η^2^.

## 4 Discussion

This study empirically demonstrated that performance deterioration in children with ADHD is primarily driven by inattention and impulsivity (37), and suggests the necessity and feasibility of a personalized DTx adjunct protocol grounded in these underlying mechanisms (38). Unlike prior ADHD digital intervention studies that have largely determined content sequencing and structure based on average treatment effects, our findings highlight the importance of precise intervention strategies that account for behavioral mechanisms and individual differences (39).

Performance accuracy in children with ADHD was more strongly associated with commission errors than with omission errors in digital training tasks, and we observed a clear speed–accuracy trade-off pattern, whereby impulsive errors increased as response time decreased. This suggests that performance impairment in children with ADHD arises not merely from insufficient attention but also from failures in impulse control, leading to premature responses (40). These findings indicate that impulsivity, one of the core symptoms of ADHD, is a critical behavioral pathology in real-world task performance. This interpretation is consistent with previous reports of increased response speed and impaired inhibitory control in patients with ADHD (41, 42). Moreover, in digital environments that frequently require rapid responses, the influence of impulsivity on performance outcomes may be further amplified (43). A key contribution of this study lies in the quantitative characterization of impulsivity-centered performance mechanisms using high-frequency behavioral data derived from personalized digital content configurations.

As the DTx adjunct training progressed, the children’s task accuracy improved significantly, while response time remained relatively stable. This indicates that the training content was not designed merely to slow down responses but to repeatedly reinforce processes that suppress impulsive decision-making and promote more stable and deliberate performance strategies aimed at improving accuracy (44). These findings challenge a common concern in ADHD digital interventions that improvements in accuracy necessarily come at the expense of slower response times and suggest that DTx adjunct training can operate in a manner that enhances self-regulatory capacity (45). In particular, our observation that reductions in impulsive (commission) errors were the primary mechanism underlying accuracy improvement underscores the importance of targeting impulse control as a core component of digital interventions for ADHD (46). The small sample size may have limited our ability to detect smaller but clinically meaningful effects, particularly for secondary outcomes such as the 22% reduction in impulsive errors which did not reach statistical significance (p = 0.140).

Furthermore, by analyzing individualized performance patterns in children with ADHD, we identified distinct user subtypes, with the effects of the DTx adjunct training being especially pronounced in a subgroup characterized by high impulsivity. The experimental group exhibited a marked reduction in commission errors, indicating that interventions tailored to baseline performance characteristics may be particularly effective in regulating impulsive responses (47). These findings also highlight the limitations of conventional approaches that assess digital intervention efficacy solely by adjusting task difficulty based on average performance changes and emphasize the necessity of analyses and interpretations that account for individual differences in ADHD treatment (48). Notably, children exhibiting prominent impulsivity may have substantial potential to acquire self-regulation strategies through digital training, and providing content and training structures tailored to this subgroup may lead to clinically meaningful improvements (49).

Based on analyses of inattention- and impulsivity-centered performance mechanisms and user subtype classification, this study evaluated the effectiveness of a personalized DTx adjunct protocol that aligns content characteristics with specific training objectives. These findings support the need to move beyond uniform digital training approaches toward differentiated intervention strategies tailored to individual behavioral profiles. DTx devices are particularly well suited for implementing personalized interventions, as they enable the continuous collection of quantitative behavioral data—such as response time, error types, and accuracy—during task performance (50). Our results indicate that such data can serve not only as outcome measures but also as evidence to inform the design and adaptive refinement of therapeutic strategies.

Furthermore, this study demonstrates the potential of DTx adjunct training as a complementary approach to pharmacological treatment for children with ADHD. In situations where non-pharmacological interventions are required due to medication-related side effects or caregiver burden, digital content-based training may represent a practical alternative in terms of accessibility and adherence (51). Moreover, the integrated use of subjective symptom rating scales, such as the K-ARS, objective performance-based assessments, such as the CAT, and digital training data enables a more precise understanding of the relationship between clinical symptom changes and actual behavioral modifications (52). This integrated framework may provide important clinical evidence for evaluating intervention efficacy and guiding treatment strategy adjustments for future ADHD interventions.

This study has several limitations. First, the relatively small sample size warrants caution when generalizing the findings. It is acknowledged as a limitation that a formal *a priori* power calculation was not performed; instead, an *a priori* sensitivity analysis was conducted to assess the adequacy of the target sample size. Second, although this study randomly compared a personalized digital intervention with a standard protocol, it did not disentangle the relative contributions of the specific components of the personalized intervention (e.g., speed–accuracy regulation and error-type-based difficulty adjustment). Future studies employing more refined designs are required to systematically examine the effects of individual personalization components. Third, the analysis primarily focused on short-term training effects; therefore, long-term maintenance effects and transfers to everyday functional outcomes could not be evaluated. Furthermore, changes in visual attention allocation and cognitive strategies during digital training were indirectly inferred through behavioral metrics. Future research integrating physiological measures, such as eye and behavioral tracking, may provide more direct insights into these processes. Due to the nature of the intervention, blinding of participants and caregivers was not feasible in this study. In addition, as the investigators were involved in administering the intervention after group allocation, full blinding of investigators was not achieved. Consequently, the possibility of expectation or assessment bias, particularly in caregiver-reported outcomes, cannot be excluded. This limitation represents a major threat to internal validity, particularly for caregiver-reported outcomes (K-ARS), as expectation bias may have influenced symptom ratings. The objective, computer-administered CAT is less susceptible to such bias. Notably, however, significant between-group differences were observed for both the CAT (Flanker Task: p = 0.024) and the K-ARS (hyperactivity-impulsivity: p = 0.025; total score: p = 0.040), suggesting that the observed effects are unlikely to be solely attributable to assessment bias. Furthermore, this study included a relatively broad age range (5–12 years), which may introduce developmental heterogeneity. Differences in cognitive development across age groups may have influenced the performance outcomes, and this potential effect cannot be fully ruled out. Due to the limited sample size, age-stratified analyses (e.g., 5–8 vs. 9–12 years) were not feasible in the present study. Future studies are encouraged to stratify participants by age group to account for developmental differences in cognitive maturation and ADHD symptom presentation when evaluating the effects of personalized DTx protocols. Future studies with larger sample sizes and randomized controlled designs are warranted to further validate the effectiveness of personalized DTx adjunct protocols. Finally, our findings may be extended to real-time adaptive therapeutic strategies that leverage digital behavioral biomarkers.

## 5 Conclusion

This study analyzed core mechanisms underlying performance deterioration in children with ADHD based on digital intervention training data, including accuracy, response time, and error types, and evaluated the effectiveness of a personalized protocol reflecting these mechanisms. The results indicated that performance impairment was more closely associated with failures in impulse control, represented by commission errors, than with inattention characterized by omission errors. A clear speed–accuracy trade-off was also identified, with faster responses being associated with increased errors. In the experimental group receiving the personalized protocol, training scores increased by 79% after 3 weeks (p < 0.001), and accuracy improved by 2.32 percentage points (p = 0.004), while response time remained unchanged (p = 0.995), suggesting enhanced performance efficiency driven by improved self-regulation rather than response slowing. In clinical assessments, the experimental group demonstrated significant improvements from baseline on the CAT Flanker task (p = 0.038, Cohen’s d = 0.93), and also showed significantly superior performance compared with the control group at 4 weeks (p = 0.024). The rate of change was likewise significantly greater in the experimental group (40.0%) than in the control group (30.9%; p = 0.040, Cohen’s d = 0.66). Furthermore, on the K-ARS, the experimental group exhibited a significantly greater reduction in hyperactivity–impulsivity scores compared with the control group (ANCOVA p = 0.025, η^2^ = 0.129), as well as a greater reduction in total scores (p = 0.040, η^2^ = 0.109), supporting the clinical utility of personalized digital interventions grounded in baseline training data.

## Data Availability

The original contributions presented in the study are included in the article/**Supplementary Material**. Further inquiries can be directed to the corresponding author.
