# Supplementary material for: Data-driven mechanistic analysis of digital therapeutic–assisted training and evaluation of personalized protocol effects in children with attention deficit/hyperactivity disorder
Source: Front Psychiatry. 2026 Jun 19;17:1794425. doi: 10.3389/fpsyt.2026.1794425 (PMC13328170; doi:10.3389/fpsyt.2026.1794425)
Supplement: Supplementary file 1 [file DataSheet1.pdf]

Chile Assent Form

# : Participant Information and Assent

The child assent form is used in clinical trials conducted with children aged 6 to 12 years..

- **Clinical Trial Title**

  A Single-Center, Prospective, Comparative, Randomized, Open-Label, Exploratory Clinical Trial to Evaluate the Efficacy and Safety of the Cognitive Therapy Software "EYAS-Focus" for Improving Attention and Concentration in Pediatric Patients with ADHD

- **Sponsor**

  InTheTech Inc.

- **Clinical Trial Site / Principal Investigator**

  Keimyung University Dongsan Hospital / Na-Young Kong, MD, PhD (Department of Psychiatry)

Some of the things we talk about today may be difficult to understand.
If you are not sure what something means, you can ask your parent (or guardian) or the doctor at any time. You do not have to decide right now whether you want to take part in the clinical trial.
Please listen carefully to what the doctor explains, and then go home and talk about it with your parent (or guardian).
When you come back to the hospital, you can tell us what you would like to do.

APPROVED
계명대학교 동산병원
DONGSAN HOSPITAL
Institutional Review Board

1. What is a clinical trial?

A clinical trial is a study that helps doctors learn whether a new medical device, medicine, or treatment works well and is safe for people.

A clinical trial is a little different from the usual treatment you receive at a hospital.

Usual treatments are methods that are already known to be mostly safe.

In a clinical trial, doctors test new medical devices, medicines, or treatments that are not yet fully known to be safe or effective.

2. Why is this clinical trial being done?

In this clinical trial, doctors and researchers want to learn more about children who have trouble paying attention.

They want to see whether "EYAS-Focus" can help you.

They will compare your results before and after using EYAS-Focus to see if your ability to pay attention improves.

3. Why are you being asked to take part?

A total of 40 children who have difficulty paying attention will take part in this clinical trial.

The doctor thinks you may be one of these children and is asking if you would like to join.

Taking part in this clinical trial may or may not help you.

Please think carefully about the information below before deciding whether you want to take part in the clinical trial.

APPROVED
DONGSAN HOSPITAL
Institutional Review Board

4. When does the clinical trial start and end?

From the day we check whether you can join the clinical trial until the last day you visit the hospital, the study will take about two months.
During this time, you will visit the hospital about four times.
The day the clinical trial ends may be different for each child.
This is because each child's situation may be different, depending on the three cases explained below.

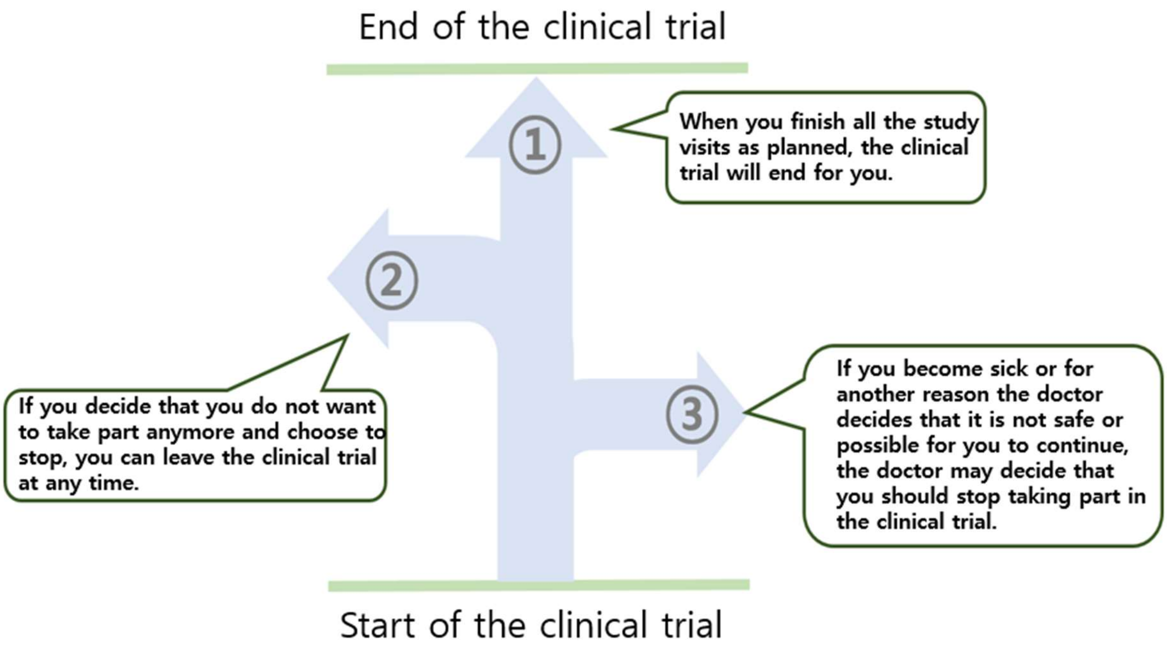

5. What will you do if you take part in the clinical trial?

If you tell the doctor or the researcher that you would like to join the clinical trial, you will be assigned by chance to one of two groups.
One group will use "EYAS-Focus" during the clinical trial, and the other group will not.

If you are chosen to use "EYAS-Focus," a researcher will teach you how to use it.
You will use EYAS-Focus at home during the clinical trial.
During this time, the researcher may ask you some questions.
They may also ask your parent (or guardian) some questions about you.

If you are chosen not to use EYAS-Focus, you will not use the software.
Instead, you will continue to take the medicine you have been using for your attention difficulties, as usual.

APPROVED
Dongsan Hospital Institutional Review Board

6. How might this clinical trial help you?

If you take part in this clinical trial and use "EYAS-Focus," it may help improve your ability to pay attention.
However, this does not mean that all children in the clinical trial will be helped by EYAS-Focus.

If this clinical trial shows that EYAS-Focus helps you, other children like you may be able to use EYAS-Focus in the future and receive help as well.

However, using EYAS-Focus may sometimes cause discomfort or make you feel unwell.
Not everyone will have these problems, but in some cases you may feel sleepy, have a headache, or feel dizzy.
If any of these problems happen, you should tell your parent (or guardian) and the doctor right away.

7. Are there other treatment options?

Even if you do not take part in this clinical trial, there are other ways to help treat you.
These include taking medicine or doing behavior therapy at home with your parent (or guardian), and other treatments.

8. What if you do not want to take part?

If taking part in the clinical trial feels scary or makes you think it might make you feel worse, it is okay not to join.
You should decide whether to take part together with your parent (or guardian).

If you decide not to take part, no one will be angry with you, and you will still be able to receive treatment at the hospital.

9. What if you want to stop later?

If you start the clinical trial but later decide that you do not want to continue, you can tell us that you want to stop at any time.
No one will be upset or angry with you for stopping.
You can always tell us if you want to stop.

The doctor will also watch your condition during the clinical trial.

APPROVED 계명대학교 동산병원 DONGSAN HOSPITAL Institutional Review Board

If the doctor thinks that taking part in the clinical trial is not good for you, the doctor may ask you to stop.
If this happens, the doctor will explain why you need to stop.

10. Will my information be kept private?

If you take part in the clinical trial, you will talk a lot with the doctor and the researchers.
The things you talk about and the tests you have during the clinical trial will be seen by the doctors and researchers involved in the study.
People at the hospital who check whether the clinical trial is safe, and people from government agencies in our country, may also see this information.

The doctor will keep the study records at the hospital for three years after the clinical trial ends.

However, your name and personal information will be kept private from everyone else.
Even if the results of the clinical trial are later published in a book or report, no one will be able to know that you took part in this clinical trial.

11. What happens if something goes wrong during the clinical trial?

The doctor will do their best to keep you safe during the clinical trial.
If something goes wrong, the doctor will try to protect you and reduce any harm as much as possible.

If you are harmed or become more unwell because of "EYAS-Focus," you may receive medical treatment, and compensation may be provided, or you may receive treatment using other methods.

12. What do I need to do?

You should keep your appointments with the hospital.
You should use "EYAS-Focus" as the doctor explains.
If you feel anything unusual at any time, you should tell your parent (or guardian) or the doctor right away.

APPROVED 계명대학교 동산병원 DONGSAN HOSPITAL Institutional Review Board

13. What if you have questions?

If you have any questions about the clinical trial, or if there is anything you do not understand after reading this, please ask the researcher or your parent (or guardian) to explain it to you.

If you have questions at any time, please contact us.

Clinical Trial Physician:

Professor Nayoung Kong, MD, PhD

Department of Psychiatry, Keimyung University Dongsan Hospital

Tel: +82-10-4145-8937

Contact for Questions About Participants' Rights:

Human Research Subject Protection Center (HRSP)

Tel: +82-53-258-6694

Human Research Subject Protection:

Tel: +82-53-258-6694

This information sheet will be copied by the researcher for you to keep.

After checking the items below, please sign if you would like to take part in the clinical trial.

APPROVED
DONGSAN HOSPITAL Institutional Review Board

[Child Assent Form]

Assent Statement

I agree to take part in this clinical trial.

1. I have read this information sheet.

2. All of my questions have been answered by the researcher in a way that I can understand.

3. I have been told, with my parent (or guardian) present, about the possible good and bad things that may happen from taking part in this clinical trial, about other choices instead of this clinical trial, and that I can stop taking part at any time.

4. I have decided to take part in this clinical trial freely, based on my own thoughts and feelings, and I agree to participate.

5. I have received one copy of this assent form.

Child Participant Date: ____________________ Name: _________________ Signature: _________________

Parent of Legal Guardian of the Participant
Date: ____________________ Name: _________________ Signature: _________________

Relationship to the Participant ____
Witness (if applicable) Date: ____________________ Name: _________________ Signature: _________________

*** Witness:**
A person who is not involved in this clinical trial and is not influenced by the investigator or research staff. The witness attends the assent/consent process and reads the assent form and any other provided documents to the participant.

Investigator
Date: ____________________ Name: ____________________ Signature: ______________________

APPROVED 계명대학교 동산병원 DONGSAN HOSPITAL Institutional Review Board
